# Localisation of Cutaneous Disease

**Published:** 1849-02

**Authors:** C. Baron

**Affiliations:** Physician of the Hospitals of Paris


*Localisation of Cutaneous Disease.* By Dr. C. Baron, Jr., Physician of the Hospitals of Paris.—The anatomical classification proposed by M. Baron, and of which we will endeavour to give some idea, has the advantage on one hand of assisting the memory of the student, and on the other of leading the practitioner to a rational mode of treatment. Dr. B. forms ten distinct classes according to the anatomical element of cutaneous disease. The first comprises the disorders of the vascular system,—roseola, measles, scarlatina, erythema, &c. Their principal character consists in redness, and in many of them the presence of feverishness shows the participation of the general circulation in the disturbance of the capillary system of the surface. In scarlatina, according to our author, it is the venous capillary net-work which is inflamed, or rather congested; the venous arterial and lymphatic elements are inflamed in erysipelas; a chronic inflammation of the superficial vascular layers constitutes pemphigus. Their preternatural development is observed in nævus, and if the deeper seated vessels are increased in size the disease acquires the nature of erectile tumours.

2. The papillæ are the organs of cutaneous sensation; their diseases will, therefore, be accompanied by increased or diminished local irritability, and the excessive pruritus of urticaria and prurigo, induce Dr. B. to class them amongst disorders of the papillary system. In elephantiasis sensation is at first deadened, and the papillæ hypertrophied.

3. Affections of the sudoriferous system are generally observed in young subjects. They are attended usually with feverishness, and either consist in augmented secretion, as in miliaria and sudamina, of inflammation of the sudoriferous organs and capillaries of the vicinity, or in the inflammation of their ducts as in herpes.

4. Maladies of the blennogenic system (secretion of epidermis) are habitually chronic and tenacious; almost in all cases the secretion of epidermis acquires an undue degree of activity; simple congestion constitutes pityriasis, inflammation, eczema, chronic inflammation, and slight induration, psoriasis; perversion of secretion, ichthyosis; increased abundance of secretion, and of hardness of epidermis, bunions, and corns.

The fifth class consists of perversion of functions of the chromatogenic apparatus. The sixth is one of the most important, comprising the maladies of the follicular system, *i. e.*, the varieties of acne and impetigo. The seventh class refers to diseases of the organs of secretion of hair, amongst which, to our surprise, we acknowledge, we find M. B. places lichen. The eighth class, which scarcely deserves that name, and might perhaps be blended in the fourth, is consecrated to diseases of the organs of secretion of nails. The ninth division has more extent and more importance. It is constituted by maladies of the fibro cellular texture of the dermis, such as ecthyma, rupia, variola, furunculus; and, finally, the tenth class characterized by the lesion of several anatomical elements of the skin in scabies.

The principle of the classification adopted by Dr. B. is undoubtedly a sound one, and we believe that it is open to fewer objections than the very numerous classifications of the same order of maladies; whilst on the other hand it possesses advantages of which they are deprived. *Med. Times.*

